# Collaboration-Centred Cities through Urban Apps Based on Open and User-Generated Data

**DOI:** 10.3390/s16071022

**Published:** 2016-07-01

**Authors:** Unai Aguilera, Diego López-de-Ipiña, Jorge Pérez

**Affiliations:** 1 DeustoTech - Fundación Deusto, Avda Universidades, 24, 48007 Bilbao, Spain; dipina@deusto.es; 2 Facultad Ingeniería, Universidad de Deusto, Avda. Universidades, 24, 48007 Bilbao, Spain; 3 TECNALIA, eServices, Velázquez 64-66, E-28001 Madrid, Spain; jorge.perez@tecnalia.com

**Keywords:** smart city, heterogeneous data, apps, prosuming, access control

## Abstract

This paper describes the IES Cities platform conceived to streamline the development of urban apps that combine heterogeneous datasets provided by diverse entities, namely, government, citizens, sensor infrastructure and other information data sources. This work pursues the challenge of achieving effective citizen collaboration by empowering them to prosume urban data across time. Particularly, this paper focuses on the query mapper; a key component of the IES Cities platform devised to democratize the development of open data-based mobile urban apps. This component allows developers not only to use available data, but also to contribute to existing datasets with the execution of SQL sentences. In addition, the component allows developers to create ad hoc storages for their applications, publishable as new datasets accessible by other consumers. As multiple users could be contributing and using a dataset, our solution also provides a data level permission mechanism to control how the platform manages the access to its datasets. We have evaluated the advantages brought forward by IES Cities from the developers’ perspective by describing an exemplary urban app created on top of it. In addition, we include an evaluation of the main functionalities of the query mapper.

## 1. Introduction

The increasing urbanisation is making city authorities, governments, companies and even citizens to start thinking about alternative ways to manage the resources within a city. This goal is achieved not only by pursuing more efficiency, i.e., to do more with less, but also by seeking to find ways to achieve a higher level of satisfaction among citizens and economic agents within a city. Indeed, the success of a city to retain and attract people and companies relies highly on its appeal, economic dynamicity and awareness of the real needs of the diverse societal sectors that populate it. Only by meeting these three premises, we can truly start talking about genuinely smart cities, i.e., “cities that meet citizens’ needs”.

Since the first public administrations started sharing their data as open data, the idea of open government has spread around the world. Open data implies [1]: (a) more efficient and effective government; (b) innovation and economic growth; (c) transparency and accountability and (d) inclusion and empowerment. These arguments favour the “open by default” paradigm, but the openness of government data is only one of the ingredients needed to address open government. involving local entrepreneurs and citizens is also necessary. Indeed, most cities, territories and countries, which have started adopting open government policies, have serious lacks on exploiting the potential of open data. They have focused their attention only on implementing their open data portals, placing low effort on bringing open data closer to entrepreneurs and citizens through suitable APIs, easily consumable by application developers.

The IES Cities platform is a CIP European project aiming to promote user-centric mobile micro-services that exploit both open data and user-supplied data. This platform contributes with an urban apps-enabling technological solution and focuses on enabling citizens to create, improve, extend and enrich the open data associated to a city, in which urban apps are based. The main stakeholders of the resulting urban apps ecosystem are the citizens, SMEs and public administration within a city. The IES Cities platform is grounded on the progress achieved lately in two key technological areas: (a) open government and urban sensor generated datasets and (b) smartphones equipped with different sensors, e.g., GPS, which can execute urban apps, i.e., offering services for citizens in different domains (e.g., transport, security and so on). Furthermore, the IES Cities platform aims to demonstrate through a range of two-phase pilots in four European cities (Bristol, Majadahonda, Rovereto and Zaragoza) how the resulting apps can actually satisfy the needs and demands of their citizens by fostering citizen-council collaboration.

In addition to presenting the global architecture of the IES Cities platform, this paper introduces the query mapper, a reusable component of the architecture that allows data consumers and producers to access the city’s datasets in a homogenous manner, independently of the initial type of the data source. This component provides a novel mechanism for accessing and contributing to the city’s dataset ecosystem. The aim of the query mapper is to allow developers to query and update datasets, which are available in different formats, using SQL sentences. This component provides developers with a mechanism for accessing static data sources (i.e., JSON or CSV), enabling them to access the data required for their applications. In addition, this component does not only allows querying data, but it also manages the update of data by creating and maintaining an internal storage that contains user contributions.

This paper has the following structure: Section 2 reviews previous work related with open data provision and management. Section 3 introduces the main characteristics of the IES Cities platform. Section 4 describes a core component of the platform, namely the query mapper, whose purpose is to streamline the consumption and production of urban data, focusing on how the component manages user provided data and its permission system. Section 5 validates the platform by describing an urban app created for Zaragoza, while Section 6 includes an experimental evaluation of the query mapper main functionalities. Finally, Section 7 draws some conclusions and outlines future work.

## 2. Related Work

In the last years, some initiatives have emerged to foster the participation of citizens in their cities through the consumption and production of urban data. This is the case of FixMyStreet [2] or FixMyTransport [3], which allow users to report problems of their surroundings and improve them. In this new paradigm, the citizen themselves gather the data, acting as sensors with their mobile devices, capturing different variables about the city and its data. Usually, citizens, with the help of sensors installed in their devices, gather and provide data related with the atmosphere (pressure, air humidity, temperature, etc.) or other data from the environment (gas emissions, pollution, noise, etc.). This data, with the one introduced into applications can be very useful to city’s public administrations and urban planners to perform improvements and interventions in the city’s infrastructure.

Data generated by citizens and gathered through crowd-sourcing, term introduced by Howe in its seminal paper [4], has been used for collecting encyclopaedia data (for example, Wikipedia [5]), cartography data (e.g., OpenStreetMaps [6]) and environmental data, as in the previous examples. The resultant datasets are useful for education, urban planning or application construction.

Our approach assembles smarter cities around the following future internet technologies: Internet of Things (IoT) [7], linked and open data [8] and crowd-sourced data [9]. IoT technologies integrate into smart cities providing knowledge about its situation and its citizens. This data sources offer their knowledge, driving it accessible to consumers and extending it with information coming from other sources or directly provided by citizens.

In this work, we propose the usage of an extendedly known query language, i.e., SQL, to enable consumers the access to data sources initially available in different formats: CSV, JSON, RDF endpoints and relational databases. Thanks to the query mapper, we aim to provide a homogeneous data access mechanism for application developers who already have some experience developing mobile applications and accessing data with a widely used language such as SQL.

The idea of using a query language to access JSON data sources is not completely new and there exist some previous examples in the literature. For example, NO-SQL stores such as CouchDB [10] and MongoDB [11] allow accessing JSON data, but they use a document-oriented approach instead of a relational one, which requires developers to understand a different access mechanism for the data. However, our approach proposes the usage of SQL, a common language for data access.

In [12] the authors propose a solution where the JSON objects are stored into rows without disaggregating them into multiple columns. However, this requires extending the SQL syntax with new constructs that enable to map the data into virtual columns. Therefore, users require learning this new constructs to query and update the JSON data. On the contrary, in [13] the authors propose a method to store JSON documents in relational system disaggregating an object into multiple rows and inserting them in a fixed structure table. As this solution stores the data aggregated inside the cells of the table, it also requires specific implementations of the SQL language to manage that specific format. Our solution, however, proposes the creation of a relational representation of the data that disaggregates the objects by creating a row per JSON object and storing its properties in specific columns. In our approach, the resulting relational representation is directly queriable using standard SQL syntax without the need to transform the queries to the internal representation, as it occurs in the previous approaches.

Apache Drill [14] is a solution that also provides access to heterogeneous data formats, including CSV and JSON, by allowing the execution of SQL sentences. However, as this proposal uses HDFS [15] for the storage of the JSON files, it requires consumers to understand the specific structure and nature of the data (e.g., internal JSON structure) to query it. Furthermore, Apache Drill does not support the execution of SQL updates, as users must insert data through the HDFS update mechanism. Therefore, unlike our proposal, it requires developers to use and understand two different mechanism for data access, instead of using a common SQL mechanism in both cases.

Our idea of simplifying the access to data sources has some resemblances with previously existing solutions that enable users to perform queries using natural language and transform them to SQL (e.g., Quepy [16]) or SPARQL (e.g., NL-SPARQL [17]). These automatic translation tools can be extremely useful for final users who do not have a technical knowledge and require exploring or even retrieving data from existing sources. Nevertheless, the usage of a formal query language (i.e., SQL), although it requires from developers to have some specific skills and knowledge, increases the accuracy when selecting data and reduces the ambiguity of and processing complexity of the open grammar introduced by natural language. In addition, the usage of these natural language translation tools does not directly provide a common access mechanism for other data source types, such as JSON and CSV, which our approach does enable to query. However, these solutions could extend our approach by providing a first layer that transforms natural language queries to SQL, easing the access for not technical users to those datasets connected through the query mapper.

On the other hand, there are several initiatives trying to promote open APIs for smart cities. For example, CitySDK [18] is a free and open-source project providing ways for citizens to release data. This SDK provides also libraries that third parties can use to reuse data released by citizens. Open311 [19] offers a platform for citizens to communicate issues about public spaces and services. It also offers a public API for third parties to develop new applications using the data provided by citizens. Besides, Open511 [20] is an open format and API to publish road event data. In the case of our solution, instead of providing an API to access a single dataset, provides a heterogeneous interface to query multiple datasets with different structures and data formats.

The publication and discovery of data sources is traditionally solved with the help of solutions such as CKAN [21], Socrata [22] or Apache Stanbol [23], being the first one the most extended solution for this problem.

CKAN is an open-source data management system, which can be used to store, manage and publish datasets in multiple formats. It provides a RESTful API for harvesting data, searching datasets and retrieving data. However, the query language used by CKAN’s DataStore lacks the expressivity of the language used in our solution (thanks to the usage of SQL) and the data storage only supports tabular data. However, the solution provided by IES Cities enables to connect to any data source in JSON, CSV or SPARQL format, allowing querying and updating of data with a sufficiently expressive language such as SQL.

On the other hand, Socrata is a proprietary solution that provides a similar solution to CKAN to access data; however, it also solves the data storage problem, providing a more integrated solution, but also sharing some of the shame limitations that CKAN has when accessing heterogeneous data. IES Cities, thanks to the query mapper, does not only allow serving new datasets but it also solves the connection with legacy data sources that already exist within the city’s ecosystem, while providing a common access and update mechanism for all the sources.

Finally, Apache Stanbol is a solution designed to connect semantic technologies within existing content management systems. However, its FactStore component lacks the tools to connect with existing data sources and focuses more on the provision of new data sources. Our solution, on the other hand, enables to provide a simpler query mechanism for SPARQL [24] data sources, which enables to bridge the gap existing among the majority of the developers and the Linked Data infrastructures.

There also exist other approaches to facilitate developers the access to open data. For example, TheDataTank (TDT) [25] is a tool to transform online data of different formats into a user-friendly Restful API. MK:Smart [26] enables the collection, integration and usage of data from diverse sources of city systems, including a data catalogue that supports the description and traceability of the data for the generation of analytics. The data integration is extended with HyperCat [27], which is a solution to integrate different IoT systems inside MK:Smart.

As in the case of our proposal, there exist other solutions focusing on providing a common and integrated access to heterogeneous data producers. FI-WARE [28] is an open initiative with the goal to create an ecosystem of ready-to use technologies, integrating sensor data and other sources. Our platform focuses on solving data access and integration issues, particularly for developers when creating their urban applications. Therefore, the IES Cities platform could be also a part of a broader solution, as it solves problems not currently addressed by other solutions when easing the access and contribution by app developers to smart cities’ data sources.

On the other hand, Ushahidi [29] represents a set of tools to democratize information, increase transparency and lower barriers for individuals to share their stories. This platform enables easy crowdsourcing of information through multiple channels, including SMS, email, Twitter and the web. It includes Crowdmap [30], a tool that enables sharing a story on a map with the addition of photos and videos. The IES Cities architecture also integrates data from different social sources accessible through the query mapper by providing a common access mechanism similar to other data sources.

Finally, this work seeks citizen participation and contribution to a city’s datasets or knowledge base by enabling them to contribute with data through their smartphones’ urban apps. Consequently, some important concerns around user-generated data are its provenance, trustworthiness and privacy control. To deal with provenance, the W3C has developed the PROV Data Model [31]. This work tackles trustworthiness by delegating data validation to the consuming app business logic but it also introduces a data access control mechanism integrated into the query and update functionality provided by the query mapper. Section 4.4 describes the flexible and fine-grained mechanism to control access to published data in IES Cities.

## 3. The IES Cities Platform

IES Cities promotes urban mobile services that exploit both open data and user-generated data in order to develop innovative services. It encourages the reuse of already deployed sensor networks in cities and the existing open government-related datasets. It envisages smartphones as both a sensor-full devices and a browser, i.e., hybrid app player, which nowadays almost every citizen carries with him.

IES Cities’ main contribution is to devise a technological platform to foster the development of open data apps, which is consumable from citizens’ smartphones. This platform is under test in four different European cities providing their citizens the opportunity to get the most out of their cities’ data. Our assumption is that urban apps will be assembled from structured and non-structured data in the form of RDF, CSV, XML or even scrapped HTML pages. However, information in such models should be mapped into JSON, a de facto lingua franca for web and mobile developers and, therefore, truly promoting the consumption of urban datasets. For that reason, the platform provides functionality to answer all user queries in JSON format, independently of the type of the underlying dataset.

IES Cities accelerates the development and deployment of new urban services that exploit city knowledge in the form of several heterogeneous datasets such as open government repositories, user-generated data through smartphone apps or even social data. The platform manages the whole lifecycle of city-related data and provides functionalities to exploit that knowledge in a uniform, web developer-friendly manner, i.e., through the SQL query language and the JSON format to exchange data between urban apps and the platform’s back-end.

Furthermore, the platform annotates user-generated data with provenance information and includes fine-grained access control metadata for datasets, enforcing quality assurance, reliability and security. The goal is to ensure that user-generated data truly enrich and complement existing council-provided data. Besides, the platform provides support to host app specific datasets so that the whole presentation and business logic can rely on the client-side while delegating all the data manipulation responsibility to the platform.

### 3.1. IES Cities Architecture

**Figure 1:**
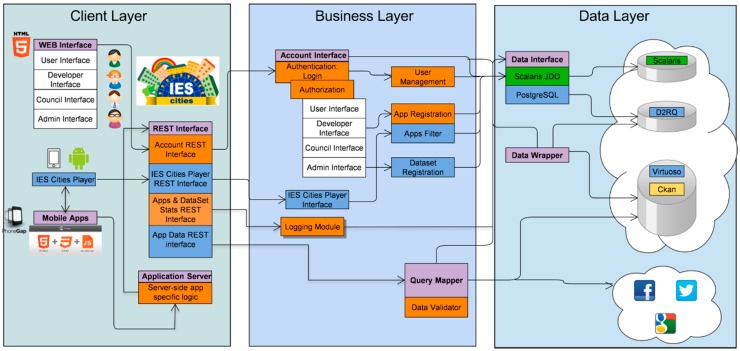
Diagram of the architecture of the IES Cities’ platform showing relations among different components.

The IES Cities platform’s client/server architecture, depicted in Figure 1, comprises the following layers: client layer, business layer and data access layer.

**Figure 2:**
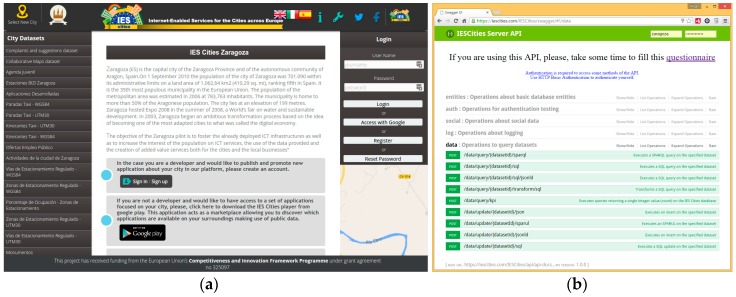
(**a**) Capture of IES Cities’ main web page; (**b**) RESTful API described using Swagger.

The *Client Layer* is composed of the *IES Cities Player* and the *Web Interface*. The IES Cities Player is a mobile application that serves as the main entry point to browse through, review and select those urban services run in a user’s smartphone. On the other hand, the Web Interface (http://iescities.com/) allows different actions depending on the type of user (citizens, city councils and service developers), e.g., to browse, search, review and access to applications, datasets and usage stats being generated by the platform. Panel (a) of Figure 2 shows a capture of this web interface.

The *Business Layer* is responsible for the management of the main entities handled by the solution, namely councils, apps, datasets, users and the gathering of usage statistics. IES Cities aims to host the urban apps and datasets ecosystem of a city. It promotes the usage and consumption of such apps and datasets by citizens, enterprises and the council itself. The platform exposes all the functionality through a RESTful API (http://iescities.com/IESCities/swagger/) that groups operations in the following categories:

- *Entities*, which offers CRUD operations to deal with the main entities tackled by the project.
- *Logging module* that enables server-side components to register diverse events associated to apps life cycle (e.g., AppStart, AppProsumer and so on), player interactions (e.g., PlayerAppSearch), or dataset-related (e.g., DatasetRegistered).
- *Social API* that provides common search methods for applications to obtain data from different social networks (e.g., Facebook and Twitter).
- *Data management* related methods, which contains methods to enable the query and insertion of data through SQL. Panel (b) of Figure 2 shows the Swagger-generated web interface that documents the IES Cities RESTful API.

Finally, the *Data Layer* allows accessing different heterogeneous datasets, e.g., ideally those modelled as 5-star linked data (e.g., rdf linked sources that are queried using sparql) but also other open data available in less rich formats such as CSV or JSON files, in an homogeneous manner. In order to make this possible, when a data provider registers a new dataset, it has to describe the contents by including a description of its data format and other related access details. In addition, the *Data Layer* also supports direct connection with different external database management systems, which contain legacy data that providers want to offer as open data.

### 3.2. IES Cities Operation

The common *modus operandi* of the platform is as follows:

1. The dataset provider (e.g., municipality) registers within the IES Cities server its datasets descriptions, by means of a web form or using an automatic process through the IES Cities API in those cases that a large number of datasets needs to be registered. For each dataset the provider indicates where the dataset can be located and accessed (URI), what is the original format of the data (CSV, JSON RDF, etc.) and, in in the case of RDF datasets, a description that correlates and maps the data from its semantic (RDF) representation to its relational view.
2. Developers discover which datasets are available by browsing or searching in the IES Cities’ dataset repository and decides which ones best fit their application. Through a RESTful JSON-based API, they obtain the description of the dataset’s structure, using a relational view of the connected data source (i.e., tables and columns).
3. After obtaining the description, the developer can start issuing queries, retrieving results in JSON format, and updates using SQL sentences. Datasets can have different permissions to access the query and update operations, see Section 4.4.
4. Once the application has been finished, developers can register it within the platform through a web form, providing among other details where the application is accessible for download (URI in the application store) and related description, including snapshots and geographical information. The IES Cities Player offers this information when showing applications to the users based on their position or their searches.
5. Finally, end-users, i.e., citizens, with the help of the IES Cities player app, browse or search for available registered urban apps according to their location and interests.

**Figure 3:**
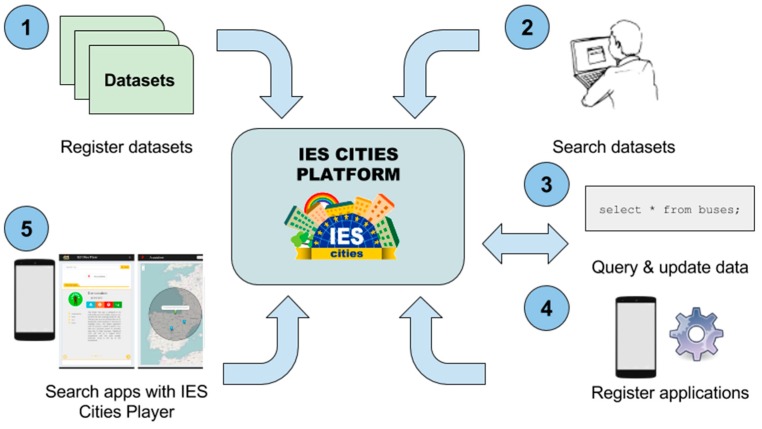
Main operation of the IES Cities’ platform showing: (1) registration of datasets; (2) search of datasets; (3) data query using SQL; (4) application registration; (5) app discovery through IES Cities Player.

Figure 3 depicts a graphical representation of the IES Cities operation including all the steps of the data publication and consumption process.

## 4. The Query Mapper

The Query Mapper component’s underlying philosophy is to create a relational view for all mapped data sources, allowing the execution of queries to retrieve an update the data. The main functionality of this module, exposed by the IES Cities API, is the following:

- *Querying using SQL.* The query mapper provides methods to query a dataset using the SQL query language. The registered dataset must be compatible with these types of queries, which requires being transformable by the query mapper to a relational view (JSON, CSV or SPARQL datasets).
- *Querying using SPARQL.* The query mapper also provides support for querying datasets using SPARQL. In this case, only datasets mapped to SPARQL data sources are compatible with this language. More experienced users can use this functionality to access data sources modelled as RDF graphs.
- *Update using SQL.* This module also contains functionality to insert or remove data through SQL from the registered datasets, thanks to the query mapper, which fully supports CRUD operations over datasets.
- *Obtain dataset’s structure.* The query mapper transforms connected data sources (JSON, CSV) to a relational storage that can be accessed (queried or updated) using SQL sentences. Therefore, developers require discovering the relational view (i.e., tables and columns) of the registered datasets, in order to start using them in their applications.

**Figure 4:**
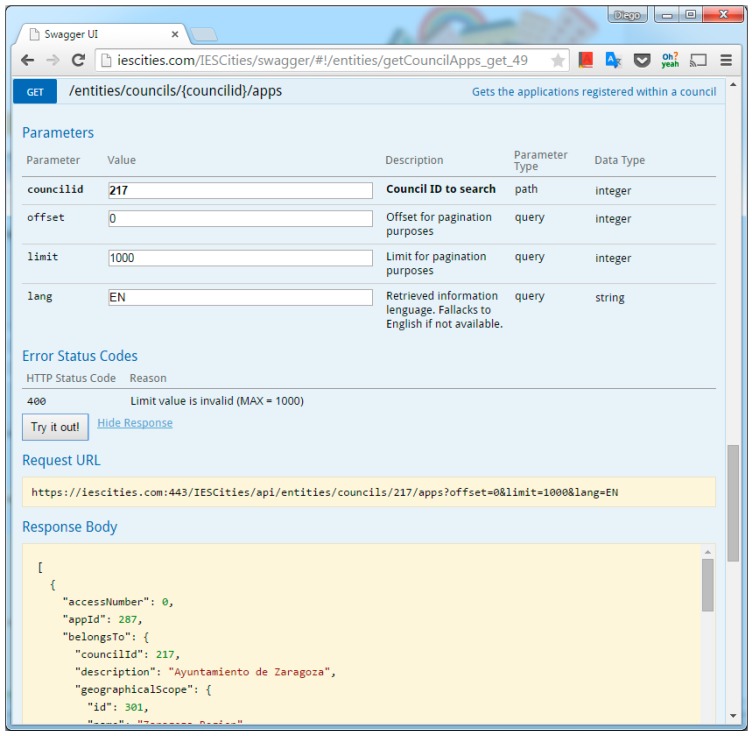
RESTful API invocation through the Swagger interface.

In addition to the previous core functionality, the query mapper also provides other utility methods in its REST API. These methods perform different functions with the data: (a) obtain the results in JSON-LD format, which is only supported for datasets containing semantic information (i.e., connected SPARQL endpoints), or perform updates in JSON format, instead of using SQL. This way, the query mapper provides a more coherent data API: as the API allows retrieving data in JSON format, it also provides methods to push data in JSON format and the query mapper will transform it to the corresponding SQL update or insert sentences. Figure 4 shows a screen capture of the invocation of a data access method using Swagger.

### 4.1. Connecting Data Sources

Datasets registered in the IES Cities platform require a *mapping* description to connect with the data sources. Mappings, described using JSON, specify different attributes, depending on the type of the connected dataset. The query mapper uses this attributes to create the relational view of the connected source. However, all mappings contain, at least, the type of the connected source. Currently, the supported data sources are the following: SPARQL, JSON and CSV, and relational databases.

**Figure 5:**
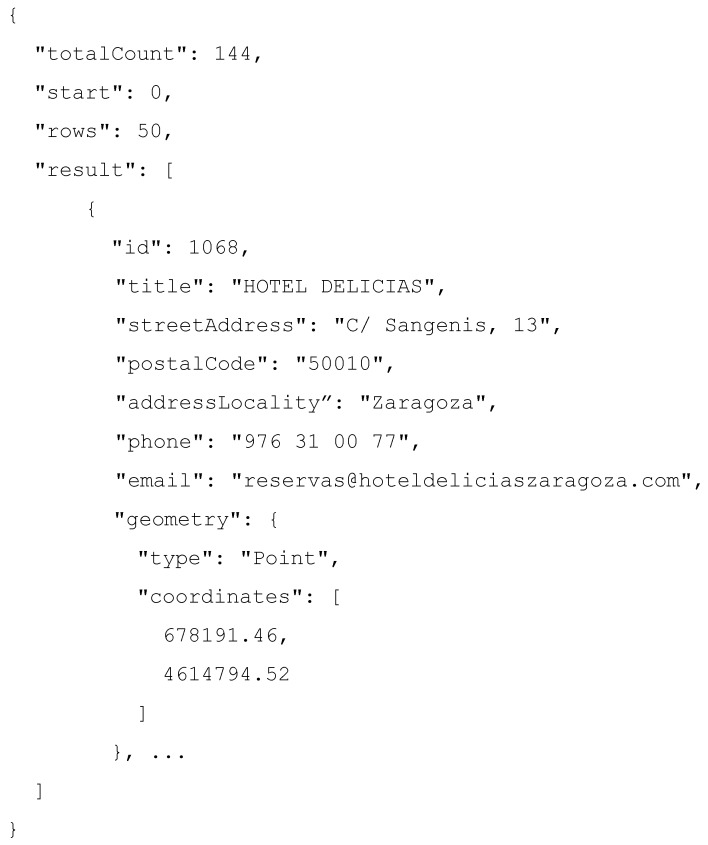
Fragment of a JSON dataset about accommodation offer in Zaragoza, Spain.

Let consider the JSON file fragment, showed in Figure 5, as an example of the mapping process. The figure shows an excerpt extracted from a publicly available data source in JSON format, provided by the Zaragoza city council, one of the partners of the IES Cities project. The full document, which contains details about accommodation options in the city of Zaragoza, is available for download at the following URL https://www.zaragoza.es/api/recurso/turismo/alojamiento.json.

**Figure 6:**
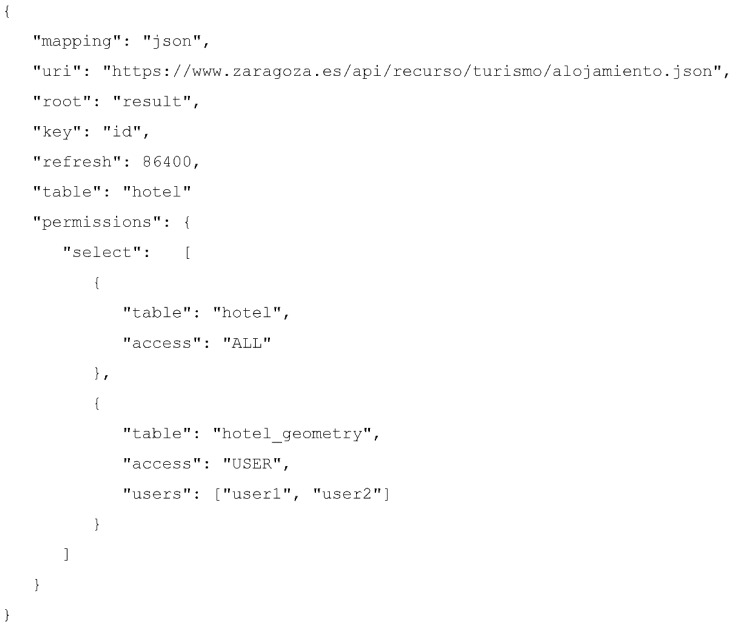
JSON mapping to register accommodation JSON dataset in IES Cities.

On the other hand, Figure 6 shows the JSON description required by the query mapper to register the previous dataset within the IES Cities platform. The description contains different attributes that provide the information required by the query mapper in order to create the relational representation of the data source.

- The mapping field instructs the query mapper that the input data source is of JSON type. Supported types are csv, json, sparql and database.
- The uri field points to the source URL where the file containing the data is located (local files are also supported).
- The root field defines the JSON key field where the mapping process starts. In the case of JSON data sources, there could exists multiple nested objects. This field allows specifying a route within the JSON data file where the data is contained.
- The key field selects the attribute from the set of mapped objects used as the primary key for the main relational table generated from this JSON.
- The refresh field defines the interval, in seconds, to retrieve the new data and update the internal structures, since JSON files can change periodically.
- The optional table field indicates the name of the main relational table generated by the mapping process. If not specified, the mapper names the table as the value of the key specified in the root field.

In the previous example, in order to give place to a 1st normal form-based relational database, the mapper will produce two additional tables from the given JSON contents, namely, hotel_geometry and hotel_geometry_coordinates. The query mapper applies this process iteratively if the JSON data file contains multiple nested object, meaning that the query mapper will create different intermediate tables as needed to represent the data in a normalized relational view.

**Figure 7:**
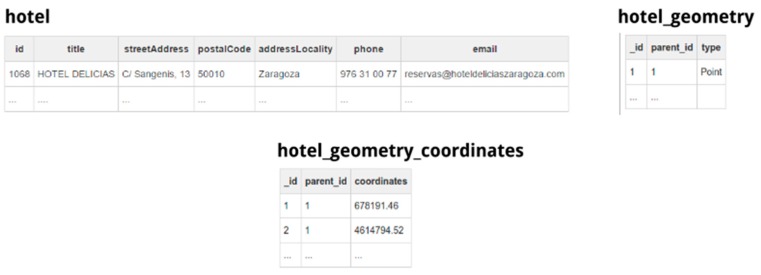
Data serialized in JSON format obtained from the execution of the SQL select statement.

The query mapper extracts the JSON dataset and creates tables representing the same data but with a relational structure. The query mapper applies an extraction procedure to create the relational view of the connected data source. Figure 7 shows the resulting table structure, obtained after applying the previous procedure to the data contained in the dataset shown in Figure 5.

The procedure used to extract the structure from a JSON object and create a relational structure works in the following way:

- The list of objects, specified by the root property of the mapping, is iterated and the query mapper converts all literal attributes (strings and numbers) to their relational equivalent representation. The query mapper applies a finer data type detection to check if those string values contained in the JSON are representing dates or, otherwise, if numeric values are representing integer or float types.
- Each object maps to a table, whose schema contains all literal properties as columns with the detected type. For example, the table hotel and the columns created to store the data of all the related objects. As in our approach the keys of the original JSON data are used as the column names of the resulting tables, we escape the name of the columns as they can contain spaces or other special characters not directly allowed as column names in SQLite. As JSON keys cannot directly include a double quote character without escaping it, the column name does not require further processing.
- For those properties that do not have a literal type but contain an array of other objects, the process creates a new child table by applying a recursive process. This child table connects to its parent using a special key column added to the child table. For example, tables hotel_geometry and hotel_geometry_coordinates, which contain intermediate data.
- If the JSON property contains an array of literal values, the process also creates a new table containing a single column, using the same foreign key mechanism explained before to connect the newly created table with the one containing the parent object. In the example, this is the case of the column coordinates of table hotel_geometry_coordinates.

The extraction process results in a 1st Normal Form (1NF) database because all related data is contained in a separate table (e.g., hotel_geometry or hotel_geometry_coordinates), eliminating repetition groups in tables (e.g., this is the case of the column coordinates in the hotel_geometry_coordinates table) and using primary keys univocally identifying each row. The mapping description allows, to the administrator that is connecting the data source, selecting which column or columns constitute the primary key of the table. If the data does not have a specific primary key for the data included in the table, the query mapper enables to create an ad-hoc numeric identifier for each column.

There is no way to ensure that the data contained in table is in 2NF (and therefore, neither in 3NF) Normal Form because it is not possible for the query mapper to extract the semantics of the table and infer which columns have a dependency relationship among them in order to define and populate new tables. Only in those cases where the original dataset contains a structure already partially normalized the process could result in a 2nd Normal Form relational database after the mapping process. This means that the query mapper cannot improve the structure of a dataset but only to provide an easier access to the data it contains through a common query mechanism (i.e., with the execution of SQL queries and updates).

The translation of the JSON structure to a 1NF Relational model disaggregates the information contained in a JSON object into multiple columns. In our approach, instead of storing aggregated data [12,13,14], we create specific tables that store each object in a different row and its properties in different columns, using the name of the keys and their inferred type to create them. Therefore, the translation results in multiple CREATE TABLE statements, one per required table, defining the name and type of its columns. Finally, the extractor populates the tables with the information contained in the JSON object. Once the process creates and extracts the data, users can execute SQL queries and updates directly on the resulting tables.

On the other hand, the permissions field manages the access control, configurable for each operation, i.e., select, delete, insert and update. The query mapper applies this control on every table provided by the relational view of a dataset. In the case of the example, for the main table hotel, everybody can select its contents; however, for the table hotel_geometry the query mapper will only allow those users identified as user1 and user2 to perform a select operation. For those operations not declared in the permission section, the access defaults to NONE, which implies that nobody can delete, update or insert contents into these tables using the RESTful API. This makes sense for this example dataset, since the council regulates it. However, for app-specific datasets, some users could have access for contributing with their information and update the dataset. Section 4.4 provides more information about the permission management feature provided by the query mapper.

**Figure 8:**
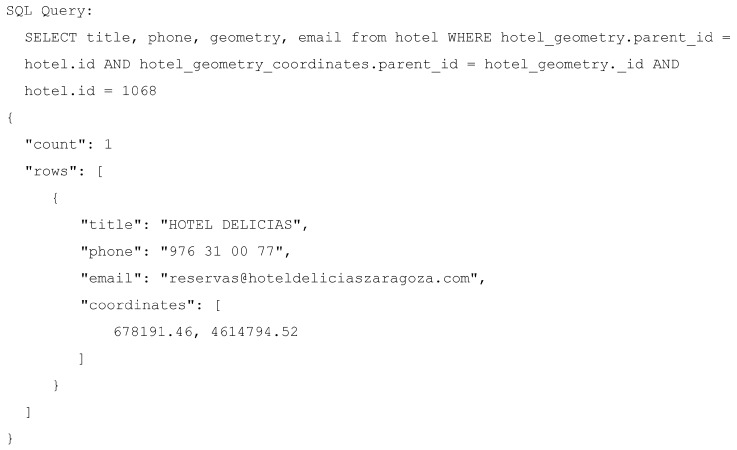
Data serialized in JSON format obtained from the execution of the SQL select statement.

Finally, Figure 8 shows the result of querying the hotels JSON dataset mapped earlier through a SQL query and the results of such query returned in JSON format.

The query mapper transforms the data into different tables in order to obtain a normalized view. Therefore, consumers could require joining them using the specific identifiers generated by the query mapper during the extraction process. For example, in this case, the intermediate tables have reference to the parent tables thanks to a column called parent_id, which was not present in the original data and it is required to join the different tables of the relational view. This way, users can obtain the information contained in the JSON structure by connecting the created tables using the intermediate columns created to store the disaggregated data. For example, in Figure 8, the query retrieves part of the information disaggregated into multiple tables. As the example shows, the query can retrieve the data with a structure different to the original JSON, i.e., the geometry JSON object is missing in Figure 8, depending on how the query selects and connects the tables and their columns.

Although, our approach requires users to manually connect the tables and it allows retrieving the data with a different structure to the original one, it provides developers a greater flexibility when selecting the specific data consumed by the applications.

### 4.2. Prosuming Data

The IES Cities platform allows users to query datasets and to contribute with data, thanks to the functionality provided by the query mapper. In both cases, the users can access heterogeneous datasets (JSON, CSV, SPARQL and relational DBs) using the SQL query language. Every time the platform connects with a new dataset, the query mapper extracts its information and creates a relational view of the data, which is, henceforth, queriable using SQL. The platform extracts the data periodically, using the configured interval, in order to maintain an up-to-date version of the extracted information for those external datasets whose source has changed.

As the data extracted by the query mapper, when connecting with JSON and CSV datasets, is backed by a SQLite database, it can solve any SQL query supported by this database management system: COUNT, DISTINCT, WHERE, JOIN, LIMIT, etc.

In our proposal, we have covered two different scenarios when users want to contribute with their own data: (a) contribution to an existing dataset and (b) creation of a new dataset from scratch. We think that these two scenarios cover the different use cases that are required for an open data platform oriented to the creation of urban applications. While the former case is useful to map and access already existing data sources, the latter allows users to easily create datasets that are specific for their applications but, in addition, that also could be useful for other consumers, if configured with query permissions for other users.

#### 4.2.1. Contribution to Existing Datasets

The contribution to external datasets depends on the type of the connected dataset. If the external dataset allows updating, the platform redirects and/or translates SQL update sentences to the corresponding format. Therefore, translation occurs with updateable SPARQL endpoints, while redirection takes place for JSON and CSV data sources transformed to relational databases. However, there exist other cases where it is not possible to update the original dataset. In these cases, the query mapper module creates an additional storage that will only contain the users’ contributions. Depending on the connected dataset type, the platform works as follows:

- *JSON and CSV datasets*. The query mapper transforms external datasets provided in these formats, as explained in Section 4.1, to a relational representation by creating a local database, one for each dataset, and downloading the data. Once the mapper creates the relational view of the dataset and downloads the data, the dataset can be queried using SQL sentences answered by the relational representation of the data. By using this approach, we are assuming that JSON and CSV datasets are not directly updateable as data servers usually provide them as downloadable files.
- *SPARQL datasets*. This type of external datasets is updateable if the endpoint is accepts SPARUL sentences. In those cases, the query mapper transforms the SQL updates to a corresponding SPARUL statement sent and executed by the endpoint. The IES Cities platform does not maintain any data and it sends all the queries and updates, once translated to SPARQL or SPARUL, respectively, to the corresponding endpoint.
- *Relational datasets*. In this case, the connected dataset is already available in a relational representation. Therefore, there is no need to translate the SQL queries and updates, but directly execute them against the connected database. Therefore, the IES Cites platform only acts as gateway to the data, which can be useful for those legacy datasets that some publishers wants to transform to open datasets. Database administrators can create relational views and/or apply the access mechanism introduced by the query mapper to control how the users access and update the data through the IES Cities platform.

In the case of JSON and CSV datasets, which are not directly updateable, user-contributed data is stored in its own internal database, one for each connected dataset. Therefore, the platform has two internal sources of information that can solve each query: one containing original information and the other one with the data contributed by the users. The user data storage has the same data structure (i.e., tables and columns) that the one containing the original data, but focused on storing users’ contributions.

**Figure 9:**
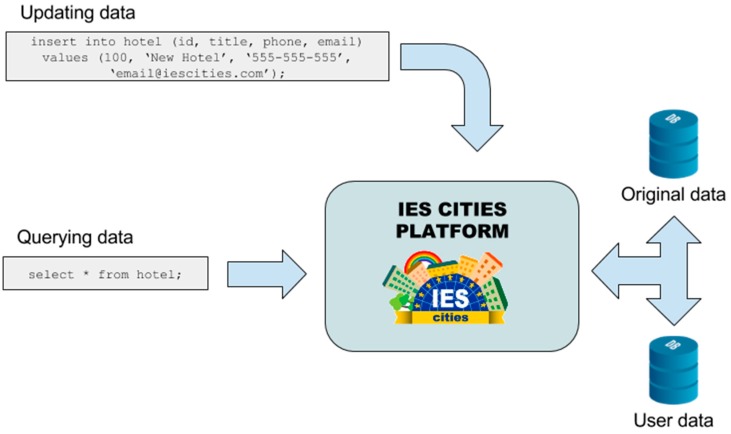
Data queries performed on the two internal storages: original data and user data.

When a user sends a query to a dataset that is split into two storages (original data and user contributions), the query mapper executes the query in both internal storages and merges the data to build a unique response for the user, as depicted in Figure 9. Furthermore, the user can select if the query executes in the original data storage, in the user contributed data storage or simultaneously in both storages. In the latter case, the query mapper merges responses obtained from the two storages into a single one and returns it to the consumer.

#### 4.2.2. Creation of New Datasets

In addition to connecting already existing datasets, the IES Cities platform allows users to create their own personal datasets by specifying their required structure. Using the dataset definition, specified in JSON, the platform creates an internal storage aimed to store data. User datasets are backed by a SQLite database file created separately for each user’s dataset. Once the Query Mapper creates the storage users can start to insert new data or retrieving already existing data using SQL sentences.

**Figure 10:**
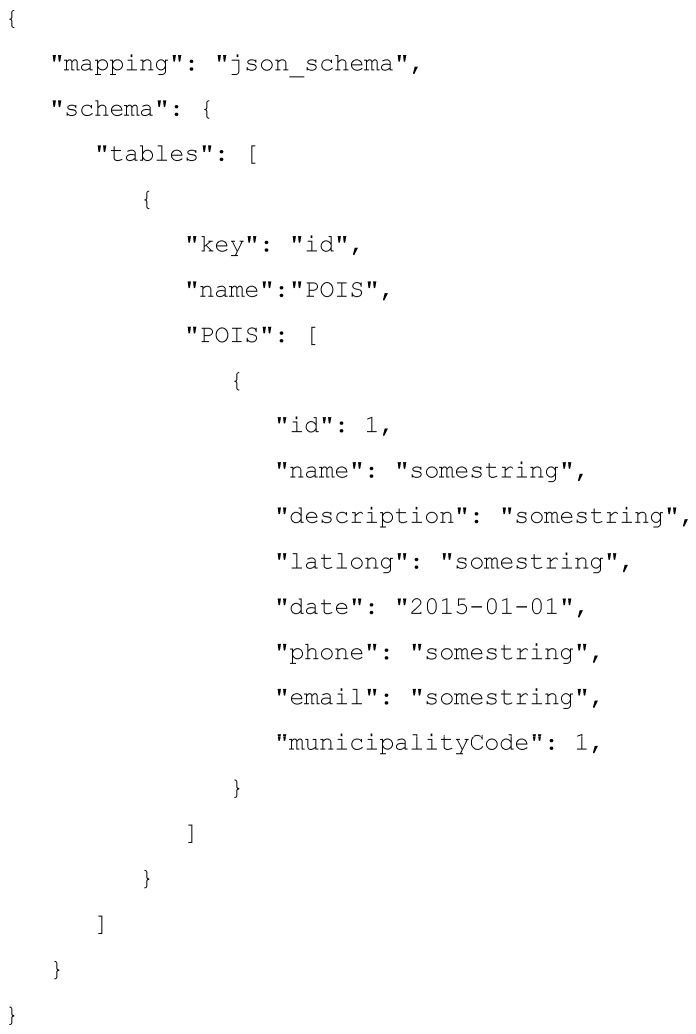
Example of user created dataset’s description.

Figure 10 shows an example of the user dataset’s structure definition. First, the JSON description instructs the query mapper that, instead of connecting with an external dataset, it must create a new internal data storage.

The description contains a list of table objects defining their structure. Each table object defines the name of the table, the key column that will be used as the primary key and a list of the table’s column names and expected values. The list of column and types is specified as pairs of field/types containing the name of the column and a placeholder for the type. For example, in the description shown in Figure 10, the name column defines its type as a string by including the somestring placeholder, while the id column defines its type as integer by including an example placeholder of that type, i.e., some integer. Finally, the date column specifies its type as a date by including a string with a compatible date format. Currently, the supported types for the data are the following: integer, float, string and date.

The creation of new datasets from their structure definition allows users storing data specifically required for their applications. Dataset owners can configure their datasets to be private or public not only for querying but also for updating. Section 4.4 describes the permission system that controls which actions are executable on a specific dataset, although the example used in this section omits the permission section due to space reasons. After creating and populating the user dataset, other urban applications could integrate user datasets, after their creation and population by their owner.

### 4.3. Data Quality

The IES Cities platform provides two quality assurance mechanisms for datasets: *quality level* and *verified by*:

- *Quality level* is a numeric property that assess the dataset conformance with one of the levels of the 5-star rating for open data (http://5stardata.info/). For external datasets, this indicator can be automatically computed from the information contained in the mapping that connects to the external dataset (JSON, CSV, SPARQL, and relational DB), which correspond with different levels of the 5-star classification.
- *Verified by* tells consumers whether the dataset and its data have been verified to fulfil the quality standards defined by its publisher or administrator. This field is useful for data consumers to decide if the dataset has the quality required for their purposes. In the case of public administrations, some manager, on behalf of the public entity, will usually be responsible for verifying published datasets.

The IES Cities platform manages dataset’s original data and users’ inserted data separately, as explained in Section 4.2. Because users’ provided data cannot always guarantee the same quality standards than the one directly provided by original sources, data separation allows data consumers to query original data and user data separately.

**Figure 11:**
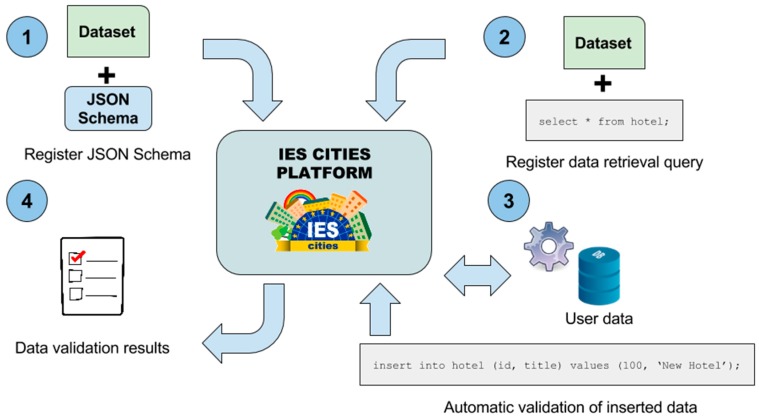
Data validation process in four different steps: (1) JSON schema registration; (2) Data retrieval query; (3) Automatic process; (4) Data validation results.

In addition, user contributed data could be integrated by the dataset administrator, after applying the proposed validation process, into the original dataset, supposing that the user generated data fulfils the quality standards defined by the publisher of the dataset. Regarding validation of datasets published within IES Cities, we have defined the following process, also depicted in Figure 11:

1. Dataset publishers provide a validation schema for their datasets. We use JSON Schema (http://json-schema.org/) as all the datasets are accessible in JSON format using the query mapper and, therefore, we can apply this mechanism to validate them. JSON Schema allows to declare the properties, data types and restrictions a JSON document must fulfil in order to be valid.
2. In addition, publishers define a query that the validator will use to retrieve the data stored in the dataset in JSON format. This query could retrieve all the dataset’s data or only a part of it for its validation, adding more flexibility to the validation process.
3. An automatic process executes the provided query on the corresponding dataset and validates the results using the associated JSON Schema, obtaining whether the data conforms to the requirements specified by the publisher or not.
4. If the data passes the validation process, the validator updates the dataset’s Verified by property accordingly. On the other hand, if the data do not comply with the specified validation schema, the administrator will need to apply some correction procedures: e.g., fix or discard erroneous data before accepting it into the main dataset storage.

The validator executes this process multiple times in order to assure the quality of the data after any change or insertion into the dataset’s storage. In addition, if configured, the insertion mechanism rollbacks any change that do not conforms to the required data schema. Using this mechanism, dataset administrators can be sure that all the inserted data fulfil a minimum data standard related with its structure and value restrictions.

### 4.4. Data Access Control

The IES Cities platform provides an access control mechanism that allows publishers to manage how users contribute to the datasets. As the users execute SQL sentences on datasets, redirected by the query mapper onto the corresponding relational view of the dataset, there are four different actions configurable on a dataset, which correspond to the usual SQL statements: SELECT, INSERT, UPDATE and DELETE. Each action supports one of the four access permissions currently supported by the platform:

- *ALL*: any user can execute actions configured with this permission.
- *NONE*: this permission expresses that users cannot execute the associated action. It is useful, for example, to create *read only* datasets, which have all actions set to NONE except for the SELECT action that can be set to ALL.
- *USER*: only users contained in the specified access list can execute the associated action. This permission can be useful for situations where only a group of users can select or update some dataset.
- *OWNER*: that only the owner of the inserted data can access or modify it, depending on the action configured for the permission.

Whenever a user contributes with new data into a dataset, the query mapper also stores the identifier of the user that inserted that information. As explained in Section 4.1, the IES Cities platform creates relational representations of the connected datasets, by downloading the associated data to an internal relational storage (JSON and CSV formats). During this process, the query mapper adds a new column to each table it creates to store the id of the user who is contributing with new data. This is the information used later by when applying the OWNER permission to an action. For example, if the permission for UPDATE and DELETE only allows access to owners, it means that users can only modify or remove those rows previously inserted by themselves and, therefore, have the corresponding user_id column set to their own user.

## 5. IES Cities-Aided Urban Apps

As part of the project, a total of 16 apps have been developed and published in Google Play (https://play.google.com/store/search?q=iescities&c=apps), ranging from apps that allow people to decide where trees are planted (e.g., Bristol’s Democratree) to collaborative maps where citizens add new points of interest and routes (e.g., Majadahonda’s In-Route).

### 5.1. Apps Evaluation Methodology

In the trialling of IES Cities’ apps, an adaptation of the Compass Acceptance Model (CAM) [32] has been taken as reference for the evaluation process. This model captures the end user feedback and performs assessment of the apps. The original CAM contains ease of use, usefulness, cost metrics and mobility evaluation factors. Ease of use and usefulness are simple to grasp as relates to the platform and apps, while cost includes factors such as effort to adopt the platform as well as actual monetary cost of the platform and applications.

To replace mobility we have added the “interaction with the city” factor. This factor measures the extent to which apps bring the citizen closer to the city and its function. We have applied the following three-step methodology to assess the degree of acceptance of the proposed urban apps by the different stakeholders:

1. Definition of a range of Key Performance Indicators (KPIs) regarding the types of users and for the different apps uses. Some common KPIs defined across apps are number of downloads, number of active users, etc.
2. Set-up of a range of data sources to feed the KPIs., which include the following:
  - User questionnaires to ask users directly about their opinion and experience with the application.
  - Logging data from logs of events generated by the apps in use.
  - Google Play, i.e., the marketplace where our apps available, to obtain usage statistics.
  - In-app questionnaires periodically launched within the apps to gather usage feedback.
3. We also have performed a mapping of data sources to KPIs. From the available data sources, we have collected and assigned values for the KPI variables.

The preliminary evaluation results obtained by applying this methodology are demonstrating that developed apps have a high degree of average acceptance (e.g., over 80%).

### 5.2. Zaragoza’s Complaints & Suggestions App

**Figure 12:**
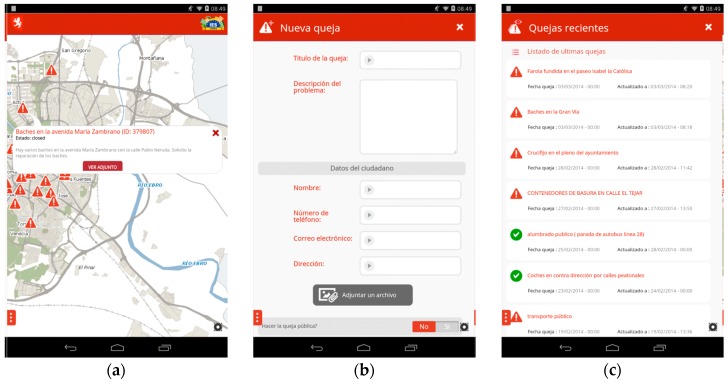
(**a**) Map showing current complaints and suggestions from Zaragoza’s city; (**b**) Form to add a new complaint to the dataset; (**c**) List of recently reported complaints and suggestions.

This app, shown in Figure 12, uses open data to get an overview of reports and faults in public infrastructure. It demonstrates how a developer with the help of the IES Cities platform can create an urban app relying on semantic data, without technical knowledge of the query and data modelling language, SPARQL and RDF, respectively. Thanks to IES Cities, a web developer only needs to create a query in the standard SQL language and send it to the query mapper, focusing on visualizing the retrieved data in their application.

Since the Zaragoza council wants to comply with standards, such as the Open311, but it also wants to commit with Open Data of the highest standards, i.e., 5* Linked Data, Open311’s GeoReport (http://wiki.open311.org/GeoReport_v2/) records have been mapped into RDF triples representing the same info in a semantic form. The application achieves this through the query mapper functionality provided by the RESTful API. Those RDF triples constitute the Open311 RDF repository made available through a simple XSLT transformation, queriable through SPARQL.

The IES Cities platform demonstrates its capabilities to accelerate urban app development by the fact that developers have only to submit SQL queries through a REST API to the IES Cities Query Mapper. This component talks to the Zaragoza SPARQL endpoint and maps the results into JSON, without requiring, for the developer, the skills and knowledge to understand the syntax of the SPARQL language.

During initialization, and after the user login, the app queries for available complaints and suggestions, and displays the result on a map, as shown in panel (a) of Figure 12. The requested information is minimal in order to reduce network usage. Users submit new complaints and suggestions by simply filling a form to which the app automatically attaches the location information, shown in panel (b) of Figure 12. On the other hand, users can review the most recent complains by selecting the corresponding menu option, shown in panel (c) of Figure 12. By clicking on the marker of a report, the app shows a description of the report and provides access to its full details.

From the data owner’s point of view, i.e., Zaragoza’s council, the enrichment of its datasets by third parties presented some issues, e.g., the fact that the app cannot publish no previously approved or the issue that there was no mechanism to control the quality and quantity of citizen added data. In order to address this, IntelliSense techniques and other consolidation techniques, combined with the one introduced in Section 4.3 in order to validate the user provided information.

## 6. Evaluation

We have evaluated the performance of the query mapper module of the IES Cities platform. Particularly, we have focus on two of its tasks: extraction of registered datasets (JSON and CSV datasets) to create a relational view, and the data access process that retrieves the stored information by executing or transforming the SQL sentences to the required format (i.e., SPARQL endpoints).

The query mapper and the IES Cities platform are implemented using Oracle Java 7 and Jersey (https://jersey.java.net) to construct the RESTful API. During the experimentation, we have used Jetty as the servlet container to serve the platform. The test platform runs an Ubuntu 15.10 on an Intel Quad Core 1.90 GHz and 8 GB of RAM. We have perform all experiments locally to minimize the latency introduced by the network during the dataset download and response transferring. This way experiments can focus on those aspects that really depend on the characteristics of the proposed mapping process and not on external factors, such as the network load. In addition, the query mapper internal scheduler, used to parallelize the dataset transformation to their relational views, uses 50 threads to run the data extraction jobs.

### 6.1. Dataset Extraction Process

**Figure 13:**
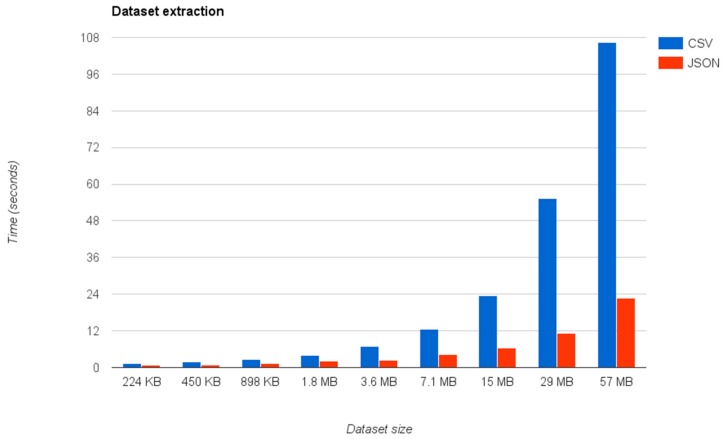
The graph shows the time required by the query mapper to extract a dataset depending on its size and its format (CSV or JSON).

The first experiment has measured how the dataset extraction process behaves under different circumstances by changing the size of the datasets and their type. As shown in Figure 13, we have repeated the experiments using different dataset sizes ranging from 224 KB to 57 MB, which approximately duplicates the size of the connected dataset in each test.

Figure 13 shows that the extraction times, i.e., the process of converting the connected datasets to their relational views is approximately 10 seconds for datasets under 7.1 MB, not only for JSON datasets but also for CSV ones. However, the experiments also show that this time largely increases for bigger datasets, especially for those available in CSV format.

The explanation for this difference in the scalability between the two formats is the way that the query mapper manages CSV datasets compared to JSON ones. In the first case, the query mapper first transforms the CSV dataset to a JSON representation, by converting the table to a list of JSON objects with the same properties contained in the CSV file. Then the query mapper transforms the resulting JSON representation to the final relational database. In the current implementation, the mapper writes the intermediate JSON file to the disk and then the JSON transforming process loads it, creating the final representation. Therefore, it means that the data extraction to the database is not a direct process. For this reason, the latency issues are going to be reduced in future implementations of the CSV extractor process by eliminating this intermediate step during the transformation.

On the other hand, Table 1 contains the total time required by the platform to extract, in parallel, multiple registered datasets. During its normal operation, the platform will extract or update the data of various datasets, using the internal scheduler of the query mapper to launch the extraction jobs. Obviously, these times will depend of the number of processor of the machine and the number of configured threads. The numbers gathered in the table show that, in the worst case scenario (500 datasets pending to be processed), the platform will finish to extract the data of all the registered datasets in approximately 20 minutes with smaller size datasets (~250 KB) and 2,3 hours in the case of bigger datasets (~3.7 MB).

Although these times can seem a bit high, we should remark that they correspond to a situation where the platform needs to process 500 datasets to extract their data. As explained in Section 4.1 the dataset mapping description contains a refresh parameter that controls how frequently a dataset requires updating. Therefore, despite a platform having a high number of registered datasets, they will not continuously require an update, meaning that the average load of the platform will be lower than the one showed in Table 1, once the system has performed the initial start-up.

Finally, Table 2 summarizes the average extraction time per dataset in the same situation shown in Table 1. As shown by the table, particularly in the case of bigger datasets, the number of configured threads and the number of available processors causes the highest processing times. However, the platform could always scale to process the extraction of a higher number of datasets if there is an increase in the number of job processors, despite any other future improve or optimization in its current implementation. In addition, the table also shows that these times are also acceptable for processing data on a platform deployed in an environment with a more limited number of datasets (up to 500) without requiring further investments to improve the hardware.

### 6.2. Querying Data

This experiment measures the total time that the query mapper requires to answer a specific query against a dataset. The complete dataset, which is a JSON dataset connected through the query mapper, contains a list of 1200 objects with 20 different properties describing each object. If an application directly consumes this dataset, it will require the developer to download and process it to extract the desired information in order to include it into the application. However, thanks to the functionality provided by the query mapper, the developer directly queries the dataset only retrieving the specific data that the application consumes.

**Figure 14:**
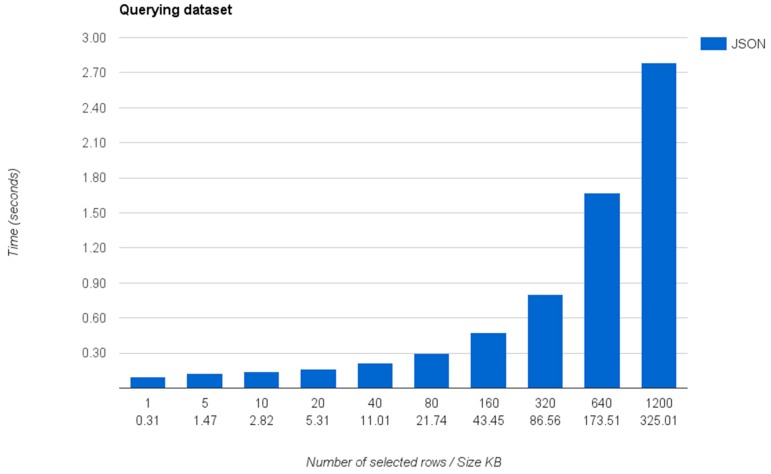
Query processing times in seconds depending on the number of rows retrieved from the dataset.

Figure 14 shows the times required by the IES Cities platform to query the data contained in the connected dataset using SQL, transform the response to JSON and received by the client.

As shown, the retrieval times are under 1 second when retrieving less than 320 rows (~87 KB) of data. The slowest retrieval occurs when the query causes the server to retrieve and transform all the data rows contained in the database (~1200 rows in the case of the example).

As observed, the inclusion of the relational representation of the dataset, currently backed by a SQLite database, introduces an overload that does not scales for a higher number of rows. We hope that this scalability issues will be solved in the future by improving the way that the data storage system is implemented and by adding a caching mechanism that allows reducing the retrieval time for subsequent queries obtaining the same response data.

We think that the flexibility introduced by the possibility to dynamically execute SQL sentences on those data sources initially only available as a static JSON or CSV file, compensates these overload issues, enabling developers to construct more easily their applications from the available city data.

### 6.3. Query Mapping Completeness

In order to show the completeness of the mapping process, we have tested the query mapper module with real datasets obtained from different open data portals. We have used 50 datasets (25 per type) and tested whether the mapping process is capable of correctly extracting all the information contained in the JSON file. Table 3 summarizes the results of the experiments. We have measured the percentage of correct relational data extractions using our proposal. As the table shows, the procedure has not been able to extract the information in all the cases. However, after inspecting the errors, we have detected that the failing extractions are due to problems in the original data sources, which provide invalid JSON or CSV files (e.g., invalid formatting, codification problems, etc.). On the contrary, in those cases that the source data is in a correct format, the extraction process has correctly extracted all the information to a relational database.

For correctly extracted data sources, we have then calculated the average number of tables generated per dataset. As it was expected, in the case of CSV datasets, the extractor generates a single table to represent the data in a queriable format. However, for JSON datasets, the query mapper generates an average number of 2.4 tables per dataset. This means that, in the random sample of datasets used for the evaluation, there is an average maximum nesting depth of JSON objects of three levels. Therefore, consumers will require creating complex queries with no more than two table JOINs.

Finally, we have compared the data retrieved from our relational mapping with the original data contained in the mapped data sources. We have executed different types of queries: simple queries that retrieve the data from the main object table, queries that retrieve and connect data from multiple tables and, in addition, queries that filter the data applying WHERE clauses. In all cases, we have compared the results obtained through our query mapper with those results expected if the same data were selected from the original data source.

The results show that, if the queries are correctly constructed (i.e., the users correctly connect the disaggregated tables using the internal identifiers) the returned data matches the original one and the JSON objects and CSV entries are correctly selected based on the applied filtering.

## 7. Conclusions and Further Work

The IES Cities platform allows councils to manage their datasets and urban apps ecosystem, aimed to increase the quality of life of their citizens and to foster economy promotion by allowing both administration provided and end-user generated data exploitation. The query mapper is the main component of the IES Cities platform that achieves this vision. This component streamlines developers work when implementing apps that consume open data and generate their own data This goal is possible by means of an SQL-based interface, which returns results in JSON, the lingua franca of web developers.

The query mapper allows connecting existing datasets (CSV, JSON and SPARQL) into the IES Cities platform and enables users to query and update data using SQL sentences. This homogenous access is achieved through the creation of a relational view that contains the same data that the original source. The query mapper connects with external data sources using a minimal mapping description, such as the format of the original dataset, the resulting primary key and other configuration aspects. Using these mapping descriptions, the query mapper automatically creates the relational version of the connected data source, allowing developers to query and update data using plain SQL sentences.

Furthermore, the query mapper also solves the problem of storing user-contributed data when the original data source is not available in a directly updateable format. This is the case of JSON and CSV data sources that are usually available as static files and, therefore, do not provide a direct update mechanism. After connecting an external data source, the query mapper component creates internal storages that allow users contributing with their own data. The component allows querying user-contributed data, interleaving it with the data source’s original but marking the returned rows as user provided in the process. Thanks to the separation between original and user-contributed data, consumers can easily identify which data was original provided by the connected dataset and which one introduced by other external contributors.

In addition, the query mapper component manages the access to the data by providing a permission system that allows to control which users can manipulate the data (query and/or modify it). This access management system enables data administrators to control those users with permission to select specific part of the dataset or to introduce new data into the user space storage. Besides, the query mapper tracks the owner of each inserted piece of data, meaning that, if required, only the user that initially specified that specific portion of the contributed data could update or remove it from the dataset.

On the other hand, we have provided an exemplary real application currently deployed in the Spanish city of Zaragoza, highlighting the properties of IES Cities to enable an easier consumption and generation of open data, and explaining how it can foster the creation of new urban application. In addition, the paper includes an evaluation showing that the process proposed to extract and convert JSON and CSV files to their relational representation behaves correctly for datasets up to ~57 MB, particularly in the case of JSON data sources. The identified scalability issues are currently related with the current implementation decisions and the characteristics of the testing environment (i.e., number of thread processors), and future releases of the platform will solve them.

As future work, we plan to add support for connecting other types of datasets, e.g., XML data sources to the IES Cities platform. However, although it could be possible to easily connect and map simple XML datasets through the SQL query mechanism, following a similar approach to one applied to JSON data sources, we need to perform further work to decide how more complex XML structures (e.g., KML [33] or CityGML [34]) can be connected and accessed through the platform. These future extensions will allow users to query and update, through the homogeneous access mechanism, a greater variety of data formats, which will enable the platform to cover the most common types for open data in urban ecosystems. Finally, in future versions of the platform, we also plan to support the execution of queries joining data from different connected data sources, aggregating data originally available in multiple formats.

## Figures and Tables

**Table 1:** Total extraction times, in seconds, when the platform contains multiple registered datasets.

Dataset Size	5 Datasets	50 Datasets	500 Datasets
232 KB	5.79	88.35	1129.09
3.7 MB	23.71	1356.89	8387.26

**Table 2:** Extraction time per dataset, in seconds, when the platform contains multiple registered datasets.

Dataset Size	5 Datasets	50 Datasets	500 Datasets
232 KB	1.16	1.77	2.18
3.7 MB	4.74	27.14	18.15

**Table 3:** Correct extraction percentage, average generated tables and query correctness per type.

Dataset Type	Correct Extraction	Average Generated Tables	Query Retrieval Correctness
JSON	93%	2.4	100%
CSV	95%	1.0	100%

## Abbreviations

The following abbreviations are used in this manuscript:

API	Application Programming interface
CAM	Compass Acceptance Model
CIP	Capital Improvement Plan
CRUD	Create, Read, Update and Delete
CSV	Comma Separated Value
GPS	Global Positioning System
HDFS	Hadoop Distributed File System
IES	Internet-Enabled Services
JSON	JavaScript Object Notation
KML	Keyhole Markup Language
KPI	Key Performance Indicators
RDF	Resource Description Format
SDK	Software Development Kit
SPARQL	SPARQL Protocol and RDF Query Language
SQL	Structured Query Language
URI	Uniform Resource Identifier
URL	Uniform Resource Locator
XML	Extensible Markup Language
